# Working from Home During Covid-19 Pandemic and Changes to Fertility Intentions Among Parents

**DOI:** 10.1007/s10680-023-09678-z

**Published:** 2023-10-17

**Authors:** Anna Kurowska, Anna Matysiak, Beata Osiewalska

**Affiliations:** 1 Faculty of Political Sciences and International Relations, University of Warsaw, Warsaw, Poland; 2 Cracow University of Economics, Cracow, Poland; 3 LabFam - Interdisciplinary Centre for Labour Market and Family Dynamics, Faculty of Economic Sciences, University of Warsaw, Warsaw, Poland

**Keywords:** Fertility intentions, Childbearing decisions, Covid-19 pandemic, Coronavirus pandemic, Working from home, Telecommuting, Telework, Home based work, Work-family reconciliation, Work-family conflict, Parents, Gender relations, Division of unpaid work

## Abstract

The Covid-19 pandemic and related massive spread of home based work led to substantial changes in the conditions for combining work and childbearing. On the one hand, working from home helped parents to accommodate increased childcare needs during the pandemic. On the other hand, it led to acute experiences of blurred boundaries between work and family life during childcare and school closures. Therefore, the direction of the impact of working from home on fertility intentions during the pandemic is not unequivocal. In this paper, we investigate how working from home was related to change in fertility intentions of mothers and fathers during the pandemic and discuss the complex mechanisms behind these relationships. With the use of unique Familydemic Survey data from a representative sample of parents in Poland, we estimate multinomial logit regressions by gender and consider a set of potential moderators, including financial well-being, gender relations, and occupational characteristics. We find evidence for an overall negative relationship between home based work and fertility intentions for mothers, but we also uncover some positive moderating effects. In particular, we shed light on the unobvious moderating role of gendered division of unpaid labor from before the pandemic.

## Introduction

The outbreak of the Covid-19 pandemic has brought a sharp decline in the TFR in late 2020 and early 2021 (Aassve et al., 2021; Sobotka et al., 2021; Wilde et al., 2020), though this negative trend reversed for some countries in the second half of 2021 (Aassve et al., 2021; Sobotka et al., 2021; UNFPA, 2021; Zeman & Sobotka, 2021). Several studies attempted to explain the mechanisms behind these developments, referring to the apparent increase in economic uncertainty (e.g., Guetto et al., 2022), job and income loss or deterioration of career prospects (e.g., Luppi et al., 2020), sudden termination of infertility treatment (e.g., Tippett, 2021) and access to external childcare (Aassve et al., 2020). No study, however, has yet looked at the role of home-based work (HBW hereafter), which became widespread during the Covid-19 pandemic, and fertility (intentions). This is quite an oversight since the massive spread of HBW has been one of the major and most universal changes that the Covid-19 pandemic has brought to workers’ lives. On average, the share of employees working from home in the EU in 2020 more than doubled compared to 2019 (Eurostat, 2021) and within the group of 18–34 year olds exceeded 50% of employees (CSO, 2021). This enormous and rapid increase in HBW together with lockdowns and school closures has completely changed the conditions of combining paid work and care during the pandemic (e.g., Adisa et al., 2021) and could have consequently affected worker’s fertility intentions, and further, their realizations.

The possibility to work from home is potentially an important determinant of fertility since it affects the conditions of combining paid work and care and career opportunities of home-based workers. On the one hand, home-based work may facilitate fertility as it may support the reconciliation of paid work and care by allowing workers to save on commuting time or organizing paid work more flexibly around family obligations (Chung and Van der Lippe 2018, Crosbie & Moore, 2004). On the other hand, however, it may also work in the opposite direction, exacerbating work-family conflict by blurring the boundaries between paid work and family life (Glavin & Schieman, 2012) and having negative consequences for workers’ career opportunities by influencing their productivity, interaction with colleagues and promotion opportunities (Kasperska, 2021; Munsch, 2016). The only two studies that have been conducted so far on the topic in the pre-pandemic period suggest that ‘the possibility to work from home at least some time’ is indeed important for fertility decisions (Sinyavskaya & Billingsley, 2015) though its influence strongly depends on woman’s family and work context (Osiewalska et al., 2022). Most importantly, HBW was found to be positively correlated with childbearing but under the condition that it was really helpful for work-family reconciliation, e.g., enabled substantial savings on commuting time or was used by those mothers, whose partners perform relatively little childcare (Osiewalska et al., 2022).

In light of these arguments, the rapid spread of HBW since March 2020 might have opened new opportunities for childbearing. At the same time, the pandemic and the related lockdowns and school closures have exacerbated numerous risks related to HBW. It has been widely demonstrated that workers who had the possibility to perform their jobs at home during the pandemic had to simultaneously take care of children during school closures, which resulted in higher mental load, lack of sleep, work interruptions and increased risk of multitasking (Adisa et al., 2021).

In this paper, we investigate how the change in access and frequency of use of HBW that took place between February 2020 and June 2021 are related to changes (increase or decrease) in fertility intentions (FI hereafter) of parents. This relatively long timespan enables us to assume that people have already had enough time to experience what HBW entails (if they had an opportunity to work from home) for work-family reconciliation, work–life balance and their professional careers during the specific, pandemic period. Therefore, our findings would not reflect peoples’ reactions to the first pandemic shock, but rather a mid-/long-term consequence of working from home during the pandemic times. Our focus is on parents of at least one child since they were able to make a full-scale experience of what it means to combine HBW with childcare and thus make fully informed changes in their subsequent fertility plans. Our study is conducted for Poland. This country in the pre-pandemic period was characterized by relatively low fertility rates (Eurostat 2019), and in particular low progression to second and higher order births, which did not reflect the desired family size of Poles (CBOS, 2019). Moreover, it also displayed rare access to and use of home based work (Eurostat 2020). This means that the studied population had a considerable potential for change in both aspects: beginning to actually work from home as well as increasing their intention to have another child (although also a decline was still possible). Furthermore, it is a country with relatively low access to childcare, low incidence of part-time work and fairly inflexible work hours (Eurostat, 2020, Eurofund 2020). Consequently, the possibility to work from home, induced by the pandemic, could have been perceived by parents as a convenient solution. At the same time, however, women in Poland bear a disproportionately high responsibility for childcare (Suwada, 2021), and thus a need to simultaneously work and take care of children at home during remote learning could have been a particular challenge for many of them. All this suggests that the opportunity to work from home might be both positively as well as negatively related to changes in fertility intentions of Poles during the pandemic.

This study has several contributions. It is the first study to explore the links between HBW and fertility (intentions) in the pandemic period. It also contributes to the scarce literature on these links from before the pandemic. It helps to better understand the role of specific circumstances for the direction of the impact of HBW on fertility decisions, both at the macro level (e.g., lockdowns, confinement measures, remote learning) as well as at micro/individual level (e.g., financial situation of the family or partner’s engagement in unpaid work). More specifically, it contributes to two major strands in the literature. First, it is the growing literature on the implications of the pandemic for fertility developments that has already pointed at the role of uncertainty (e.g., Guetto et al., 2022), job and income loss or deterioration of career prospects (e.g., Luppi et al., 2020), sudden termination of infertility treatment (e.g., Tippett, 2021) and access to external childcare (Aassve et al., 2020) for fertility decisions, but has not yet explored the role of HBW. Furthermore, we also contribute to the literature on the impact of HBW on fertility in general. Past research examined numerous consequences of this work arrangement for workers’ life, such as work-family balance, psychological well-being and health (Demerouti et al., 2014; Gajendran & Harrison, 2007), time use (Powell & Craig, 2015), working conditions or work careers (Arntz et al., 2019; Chung & van der Horst, 2018), but has rarely explored fertility (intentions) as an outcome so far. Exploring the role of HBW for fertility decisions is particularly important as the expansive use of HBW may not end with the Covid-19 pandemic, but become a new standard or at least an option for a substantial share of employees (ILO, 2020). The consequences of HBW on people’s lives may thus be widespread, long lasting, and understanding better the conditions that foster positive or negative effects of HBW on fertility decisions surely deserves special research attention.

## Theoretical Framework and Hypotheses

### Working from Home and Fertility

The most straightforward mechanism behind the influence of HBW on fertility decisions are the opportunities this work arrangement creates for combining paid work and family. These opportunities can be particularly appreciated by parents if they have the possibility to work from home and experience what it means for combining paid work and care. Parents may increase their fertility intentions if they are able to work from home as HBW may help to relax time constraints, reduce commuting times and allow more time to be devoted to family life (Chung and Van der Lippe 2018). Working from home may also allow working parents to organize paid work around childcare and housework which would not be possible if they would work from the office, i.e., to perform paid work in parallel to some household tasks (e.g., laundry or cooking) after initiating them (Bailey & Kurland, 2002; Hill et al., 2003), when children sleep (Chung and Van der Lippe 2018, Powell & Craig, 2015) or are old enough to manage on their own without supervision (Callister and Singley, 2004). Qualitative studies suggest that women who work from home choose this work arrangement to accommodate paid work and family demands (Hilbrecht et al., 2008; Sullivan & Lewis, 2001). Some studies also show that HBW may lead to higher work–life balance (Crosbie & Moore, 2004; Felstead et al., 2002). What is more, HBW brings material gains too. People who work from home can save money for some future child-related expenses, which they would otherwise spend on transport or office dressing (Madsen, 2003; Raiborn & Butler, 2009).

But HBW may also have negative effects on the work-family nexus and thus negatively impact fertility (intentions). First of all, HBW may exacerbate the work-family conflict, by blurring boundaries between paid work and family life and experiencing higher paid or unpaid workload (Glavin & Schieman, 2012; Kurowska, 2018). No clear setting of the beginning and the end of the working day and no physical boundaries between the workplace and the home may result in the negative spillover from one sphere to the other (Glavin & Schieman, 2012; Lott, 2018). Studies showed that HBW can lead to longer working hours (Chung & Van der Horst, 2018; Eurofound & ILO, 2017), more multitasking and time fragmentation—particularly among women (Hill et al., 2003; Powell & Craig, 2015)—and (as a result) higher mental load (Eurofound, 2020; Gadeyne et al., 2018). All this creates unfavorable conditions for making childbearing decisions and may negatively impact fertility intentions.

All in all, the overall effect of HBW on fertility intentions depends on the circumstances, which may foster the dominance of either positive or negative effects or cancel both effects out (Osiewalska et al., 2022). Looking first at the overall specificity of the pandemic, we argue that the lockdowns, school and childcare closures followed by widespread moves toward remote learning, even for the youngest children, as well as frequent individual and familial quarantaines created a situation in which the negative effects of HBW on the work-family reconciliation by parents dominated (outweighed the positive ones). People were faced with the need to simultaneously combine paid work and childcare or/and homeschooling at their homes. This resulted in an immense increase in unpaid tasks, work fragmentation, multitasking and mental load (e.g., Hjalmsdottir et al., 2021; Raile et al., 2021). Limited possibilities of outsourcing not only childcare but also housework (due to confinement measures) created additional burden on families. Mothers were the ones to bear the most of it (see, e.g., Meraviglia & Dudka, 2021; Zamberlan et al., 2021; Manzo & Minello, 2020), but fathers have also increased their engagement in unpaid work during the pandemic (see, e.g., Derndorfer et al., 2021, Farre et al., 2021). These experiences have been shared by large parts of the population across all countries. But they were particularly pronounced in Poland due to comparatively longer periods of time that children spent at home due to pandemic-related school closures (see Kurowska et al., 2023). These considerations lead us to an expectation that in contrast to the overall positive effects of HBW on fertility intentions (Sinyavskaya & Billingsley, 2015) or no main effects on second births (Osiewalska et al., 2022) found in studies conducted before 2020, we will find overall negative effects of HBW on fertility intentions among mothers as well as fathers (although to a lesser extent among the latter) during the coronavirus pandemic. In other words, we expect that during the pandemic the negative effects of HBW for fertility intentions among parents would—on average—dominate over the potential positive effects that HBW could have. Therefore, the first hypothesis we formulate is the following:

#### Hypothesis 1

The overall relationship between HBW and fertility intentions among Polish parents during the Covid-19 pandemic was negative (**H1a**), at least among mothers (**H1b**). In other words, we expect that fertility intentions of parents (mothers in particular) who worked from home declined more strongly or increased less strongly than fertility intentions of their office-based counterparts.

### Moderating Role of Changes to Financial Situation of the Family

Numerous confinement policies that accompanied distortions to childcare during the Covid-19 pandemic (particularly during lockdowns), led not only to the widespread use of HBW but also increased employment and income instability (Brugavini et al., 2021, Fana et al., 2020) and thus put (additional) economic strain on families. For those parents who suffered financially during this period, HBW might have been perceived as a particularly convenient working arrangement that helped them save on commuting and office dressing and thus resist the economic hardship caused by the pandemic and accommodate future child-related needs. It has been shown that money savings have been one of the important advantages of working from home for people during the pandemic (Kučera et al., 2021, Rubin et al., 2020) and that financial savings from HBW were indeed substantial (Beno, 2021). In Poland, where living standards are comparatively lower than in Western countries, these financial savings may have held particular significance for families. We therefore expect that:

#### Hypothesis 2

Among Polish parents whose financial situation deteriorated during Covid-19 pandemic, HBW might have brought enough positive gains that canceled out or even outweigh the negative effects of HBW on fertility intentions during the pandemic. Therefore, we expect to find no negative effect, or even positive effect of HBW among parents whose financial situation deteriorated during the pandemic.

### Moderating Role of Gender Division of Unpaid Work Prior to the Pandemic

In our main hypothesis, we expect to find a negative relationship between HBW and fertility intentions among mothers, as they were the ones to bear the most of the additional unpaid work during the pandemic (e.g., Sevilla & Smith, 2020). However, for those mothers, whose partners had already been involved in sharing childcare duties before the pandemic, the increase in unpaid work during the pandemic was likely smaller than for mothers who shouldered the majority of childcare responsibilities before the pandemic. It has been shown that in countries with more equal division of labor, women were less burdened with pandemic-related unpaid work than in other countries (Del Boca et al., 2021) as their partners took over part of the new responsibilities which emerged with the closure of childcare centers and schools. Lower overall increase in unpaid work—childcare in particular—while working from home would likely mean weaker negative effects on fertility intentions among women living in more egalitarian partnerships. We thus expect that:

#### Hypothesis 3A

The negative impact of HBW on fertility intentions during the pandemic was weaker among Polish mothers who shared childcare more equally with their partners already before the pandemic than among mothers who were fully/mostly responsible for unpaid work.

At the same time, however, it could be argued that women who live in egalitarian or nearly egalitarian relationships may be used to the situation in which they share childcare duties equally with their partners or at least receive substantial support from them. They may even have more demanding jobs than other women, which simply do not allow them to spend much time or energy on childcare and/or housework. Thus, a sudden increase in childcare-related duties, which they had to carry out during the pandemic while working from home, might have discouraged women living in egalitarian relationships particularly strongly from thinking about enlarging their families. This might have been the case even if their partners took over some of the additional childcare and housework. Numerous studies have shown that the additional housework and childcare, which emerged during the pandemic, was primarily carried out by women, regardless of whether they previously lived in egalitarian relationships or not (Hank & Steinbach, 2021; Manzo & Minello, 2020; Meraviglia & Dudka, 2021; Zamberlan et al., 2021). Mothers from more traditional families, in contrast to those from more egalitarian ones, might have been more used to the situation in which they have to carry out most of the childcare and housework and could more easily accept an increase in family-related responsibilities without questioning it (see, e.g., Sullivan & Lewis, 2001; Bailey & Kurland, 2002; Hilbrecht et al., 2008). For them, the possibility to work from home could emerge as a convenient solution for combining paid work and care. In fact, a study by Osiewalska et al (2022) from the pre-pandemic period in the UK showed that HBW is more likely to increase fertility of women who are primarily responsible for childcare. We thus formulate a competing hypothesis to the previous one:

#### Hypothesis 3B

The negative impact of HBW on fertility intentions during the pandemic was stronger among Polish mothers who shared childcare more equally with their partners already before the pandemic than among mothers who were fully/mostly responsible for unpaid work.

### Moderating Role of Occupational Characteristics

Finally, while for some women, work may be perceived as a parallel career to childbearing, for other women it may seem as an alternative life path to employment, particularly temporarily, when their jobs are unsatisfactory. Consistently with the New Home Economics (Becker, 1993), resigning from an unsatisfactory job in such circumstances may imply low opportunity costs and enlarging the family size may provide a woman with better self-fulfillment and in fact higher satisfaction (Friedman et al., 1994). Lockdowns and confinement policies during Covid-19 pandemic have enforced HBW across different sectors, branches and occupations. But not for all occupations working from home is a convenient/suitable working arrangement. While for managers and professionals working from home is amenable (and thus these occupational groups had the highest proportion of workers reporting doing at least some usual hours from home already before the pandemic), for other occupational groups working from home may not be equally convenient (Dockery & Bawa, 2020; Holgersen et al., 2021). Professional and managerial positions are characterized by high levels of job autonomy, in contrast to other occupational groups. And according to a meta-analysis by Gajendran and Harrison (2007) the effect of HBW on job satisfaction is to be fully mediated via autonomy. At the same time intensity/frequency of HBW may also have an impact on job satisfaction. Extensive use of HBW can increase isolation and frustration, which in turn leads to lower job satisfaction (Golden, 2006; Mergener & Mansfeld, 2021). Referring to the effect of HBW intensity/frequency on job satisfaction Golden and Veiga (2005) argue that with little autonomy, the increase in job satisfaction for low levels of HBW intensity would be weaker, while the decrease in job satisfaction for higher levels of HBW intensity would be stronger compared to HBW users with more autonomy. Therefore, we can expect that for non-managerial and non-professional workers frequent and prolonged HBW arrangement could have been particularly detrimental for job satisfaction. For mothers, holding jobs characterized by low autonomy, which do not profit them or are not suited to be executed from home, being stuck at home working intensively for a long period of time and combining this effort with childcare might have resulted in significant drop in their work satisfaction and lead to increased fertility intentions with a hope to temporarily opt out from working actively through subsequent childbearing. In Poland, such a strategy could be particularly tempting as Poland offers relatively long (up to 52 weeks) and generous maternity/parental leave entitlements (leave benefit covering between 100 and 80 percent of earnings, depending on the length of leave) for nearly all working women (Kurowska et al., 2022). Furthermore, employment of pregnant women, as well as women on maternity/parental leaves is protected (employees cannot be fired while pregnant or on leave). As a result of these considerations, we expect that:

#### Hypothesis 4

Among Polish mothers holding non-professional/non-managerial positions, prolonged, frequent work from home will be positively related to fertility intentions.

#### Country Context

Our study is located in Poland which has been the lowest low fertility country for more than two decades with the total fertility rate oscillating between 1.3 and 1.4 throughout the 2000s and 2010s. Low fertility has been largely driven by low progression to second and higher order births (Sobotka and Fuernkranz-Prskawetz, 2020, Rossa & Palma, 2020). It persisted despite the fact that the economic recession, which took place in Europe between 2008 and 2012, was relatively mild in Poland and that the country entered a track of fast economic growth in the following years. While other post-socialist countries experienced substantial improvements in their fertility rates, the TFR in Poland hovered below 1.4 till 2016 when it rose to 1.48 to start declining gradually in the following years.

Household financial needs are one of the important reasons for this persistence of low fertility. Low salaries, insufficient for purchasing a larger apartment and covering childcare-related expenses, have been repeatedly enumerated as important barriers to family formation (Marczak et al., 2018; Suwada, 2019). The introduction of generous family transfers in 2016, under the “500 + Programme”, might have eased some of these financial tensions. The program complements the heavily means-tested family benefit, which is only granted to families in the highest need, with a universal cash transfer of 500 PLN (around 120 EUR) paid monthly for each child in the family (Lendvai-Bainton & Szelewa, 2020). Nonetheless, economic activity remains one of the most important sources of income for families (GUS, 2021). Importantly, women's earnings substantially improve households’ economic situation: the disposable income of the single earner household constituted only around 60% of the income of the dual earner household (Osiewalska, 2019) and the proportion of female breadwinner couples in Poland is one of the highest in Europe (Vitali & Arpino, 2016).

Despite the fact that women’s economic activity may substantially improve households’ financial situation and thereby improve the conditions for family formation, the conditions for work and family reconciliation in Poland are very poor (Matysiak and Węziak-Białowolska 2016) and were often mentioned as an important barrier to partner’s reproductive choices (Kotowska et al., 2008, Mishtal, 2012). Poland has one of the lowest enrolment rates in creches and kindergartens among the OECD countries, with only slightly more than 10% of children below 3 attending creches just before the pandemic (OECD Family Database 2022). In the absence of places in childcare centers parents often benefit from the support of their relatives, mostly grandparents (Bordone et al., 2017). At the same time, it is uncommon to combine childcare with part-time employment. Until the pandemic employees had relatively little flexibility when it comes to the organization of their working time or working from home (Eurostat, 2020). Specifically, in 2019, i.e., just before the outbreak of the pandemic, less than 10% of employees in Poland worked from home at least occasionally, while in Nordic countries this proportion was already exceeding 25% (Eurostat, 2020). On top of that, Poland is characterized by a relatively traditional division of childcare duties: while social surveys consistently show that Polish women are expected to work for pay, they are also deemed mainly responsible for either providing or organizing childcare (Boehnke, 2011).

In these circumstances, the possibility to work from home, which emerged during the pandemic, could, on the one hand, become an important solution for combining paid work and care for some Polish mothers and increase their fertility intentions. On the other hand, however, the pandemic disorganized childcare arrangements of many parents and made combining paid work and care much more difficult. Those parents who received childcare support from grandparents suddenly had to give it up to protect the health of older family members. Moreover, access to childcare facilities became more difficult. All childcare facilities were fully closed during the first three months of the pandemic (March–May 2020; see Table 4 in the Appendix). This changed in June 2021, but the facilities for the youngest children, below 3, were opened only partially and it was up to the director to decide how many children could be admitted to the childcare center and at which hours so that the social distancing rules were respected. Schools, in particular for children aged 10 + , remained closed for most of the time and children had to attend classes remotely (see, e.g., ECDC, 2022 and Table 4). During the entire period under the analysis, any Covid-19 cases which were reported resulted in sending all the children from the class or kindergarten group into a quarantine which lasted 10–14 days. Children who displayed any symptoms of sickness—such as a running nose—which would usually go unnoticed, were asked to stay home. Parents of children aged 8 or less were offered a care allowance at 80% of their earnings if the childcare center was closed and the parent had to take time off from work in order to take care of the child (ECDC, 2022). Because of the school closures, both parents reported spending more time on childcare, though women (33%) more often than men (21%) (Own computations based on Polish Familydemic Dataset).

## Data and Research Sample

In order to investigate the links between HBW and fertility intentions during the pandemic, we make use of unique, representative data from the Polish Familydemic Survey (see Kurowska et al., 2023). The data were collected in June 2021 on a sample of women and men aged 20–59 drawn from the National Research Panel Ariadna in Poland (*Ogólnopolski Panel Badawczy Ariadna; hereafter OPBA*), which hosts over 150,000 active panelists aged 15 and over, with verified profiles. The OPBA holds a certificate from the Interviewer Quality Control Programme (*Program Kontroli Jakości Ankieterów*) and works according to the standards of ICC/ESOMAR International Code on Market and Social Research. Respondents in OPBA are given points for completing online surveys which further may be exchanged for gifts. We have used quota-random sampling, i.e., random samples with additional quotas applied to secure adequate representation of the population in the sample by crucial socio-demographic characteristics (age, gender, education and area of residence). In case of non-response, another person was sampled fulfilling certain socio-demographic characteristics. A total of 11,183 respondents aged 20–59 completed the online survey, of which 4,188 were parents of children aged under 12.

The Polish Familydemic Survey collected comprehensive information on the lives of respondents and their families over the time period starting just before the outbreak of Covid-19 till the time of the interview. Among others, it provides detailed information on the socio-economic characteristics and health status of respondents and their partners, partners’ performance in the labor market before and during the pandemic with detailed information on whether the person had the possibility to work from home (every day or occasionally), partners’ division of childcare and housework, availability of external childcare, time spent by children out of school / in remote schooling, data on respondents’ attitudes toward work and family, gender role ideologies, satisfaction with family life and relations with the partner and obviously partners’ fertility intentions pre-Covid and at the survey time. Having this rich information, we were able to investigate the links between HBW and fertility plans after accounting for the fact that the pandemic turned many other aspects of respondents’ lives upside down. This includes, among many, sudden changes in health status of respondents and their family members, labor market situation and ways of working, increase in difficulties in combining paid work and care, experience of school closures or difficulties with arranging external childcare.

To study the change in fertility intentions of mothers and fathers during the pandemic, we focus on respondents at reproductive age (20 to 44 years old; the initial sample size of 3,388 women and 2,563 men). We select only those who were in heterosexual unions at the interview, as their fertility intentions are the closest to be realized and thus the most vulnerable to the change in external conditions caused by the outbreak of Covid-19 (2,601 women and 1,837 men). As we are interested in working arrangements (HBW), we further select those who were employed both before the pandemic and at the interview (1,344 women and 1,283 men). Furthermore, we excluded those respondents who were pregnant or whose partner was pregnant at the interview (around 5% of women and men). We did not exclude couples who have a newborn child born between February 2020 and June 2021, instead we control for these situations with the use of the age of the youngest child. We checked, however, that our results are robust on the inclusion of these individuals in our sample (results available on request). Lastly, we selected only those who already have at least one child and who provide complete information on our variables of interest so that the final samples amount to 814 mothers and 877 fathers.

## Method

Our response variable is built based on two questions on fertility intentions, from which one relates to the pre-pandemic times: ‘*Did you intend to have a child within the next 3 years before the outbreak of the pandemic (February 2020)?’*, and one concerns the current situation: ‘*Do you intend to have a child within the next 3 years?’*. The answer ranges from 1—‘*definitely not’* to 10—‘*definitely yes’*. Then, we measure the change in fertility intentions comparing current intentions with pre-pandemic intentions. These changes range from -9 for the highest decrease in fertility intentions to 9 for the highest increase. As such, all the negative values stand for the decrease in childbearing intentions, 0 stands for ‘intentions hold the same’, and all the positive values represent the ‘increase in fertility intentions’.

Our key explanatory variable related to HBW is the change in access and frequency of use of HBW that took place between February 2020 and June 2021. The information on whether the respondent has only gained access to HBW during the pandemic we derive from two questions: 1) *‘Did you have an opportunity to work from home before the outbreak of the Covid-19 pandemic? (February 2020)’*, and 2) *‘Do you currently have an opportunity to work from home?’*. The information on (frequency of) actual use of HBW before the pandemic and at the moment of the interview we acquire by using two other questions: 1) *‘How often did you work from home before the outbreak of the Covid-19 pandemic?’* and 2) *‘How often do you currently work from home?’*. Based on these questions, we identify people who: a) had no access to HBW prior the pandemic and still have not got it by the time of the interview (‘*no access—no access’* category; 62% of our sample); b) those who had no access to HBW prior the pandemic but got it during the pandemic (*no access—access*; 14% of the sample); c) those who had access to HBW already but haven’t changed the intensity of its use during the pandemic (*access—access*; 17% of the sample); and d) those who had access to HBW before the pandemic but only during the pandemic took advantage of it or intensified its use (*access—access* + ; 7% of the sample). As the last category was smaller in size, it was collapsed with the third category (*access-access*) for further analysis. Respondents who have lost access to HBW during the pandemic were excluded from further analysis as they were too few to construct a separate category.

Using a multinomial logit model, we regress the change in fertility intentions (*decrease, increase, holding the same*) against our main explanatory variable, i.e., change in the access and use of HBW. We conducted all computations using the R programming language and utilized version 1.1.3 of the *ggeffects* package to determine predicted probabilities of reduced and elevated fertility intentions.

As formulated in our hypotheses 2–4, we expect that the relationship between the change in FI and HBW access/use may be moderated by certain conditions. These include: worsening of the financial situation during the pandemic, the division of childcare in the family before the pandemic, and holding a non-managerial/non-professional occupation. We test the hypotheses related to these three covariates by interacting them with our main explanatory variable. We run our models separately for mothers and fathers.

We measure the division of childcare duties between partners by an index built based on questions: *Who in your household did the following childcare tasks before Covid-19?*. These tasks include physical care (e.g., bathing, feeding, putting to bed), playing/reading, helping with schoolwork, transport and accompaniment to activities, and general oversight and supervision. We then sum up the number of tasks which women do *always or usually*. As such, the childcare index takes values between 0 (equal division or a man does more) and 5 (a woman does all childcare). Second, the worsening of the financial situation is identified based on a choice of a statement: *Comparing the current situation with the month before Covid-19 the financial situation of my family somewhat deteriorated / deteriorated a lot*. Finally, occupation is identified using answers to a question: *What is your (main) occupation?* and coded using ISCO-08. To address our fourth hypothesis, we build a dummy variable where 1 indicates non-managerial or non-professional positions.

We account for many other changes in one’s life related to both professional and family spheres that may influence childbearing intentions and the HBW status and thus confound the relationship between these two variables. We consider the change in partnership status (getting married) and worsening partnership quality (*Comparing the current situation with the month before Covid-19 my relationship with my partner: somewhat deteriorated / deteriorated a lot*). We also account for the duration of the use of HBW during the pandemic by the partner of the respondent (in months). Further, we control for Covid-19 health risk for a respondent and other household members (*Do any members of your household have a health condition that puts them at higher risk of poor outcomes from Covid-19?*). Finally, we control for housing conditions that are particularly important during the pandemic (*How sufficient is your housing for working from home or homeschooling?*), socio-economic status (educational level, difficulties to maintain the family on present HH’s income), and age (20–24; 25–29; 30–34—reference category; 35–39; 40–44). We additionally control for the number of children and the age of children (0–14 months; 15–35 months; 3–6 years; 7 years or older).

## Results

### Descriptives

Among the 1,700 respondents selected for our analysis of fertility intentions in Poland, 42% of women and 33% of men had access to HBW in June 2021. This represents an increase of 15 percentage points among women and 12 percentage points among men compared to the pre-pandemic period (see Table 1). Of those who had access to work from home during the pandemic, 27% reported that their partners also had access to HBW. More than 12% of women and 9% of men reported a decline in their fertility intentions as compared to the pre-pandemic times (Table 2). For another 9% of women and 11% of men, fertility intentions increased in the analyzed period. The majority of those with increased fertility plans are at young reproductive age (20 to 34). The decrease is pronounced among those women and men who gained access to HBW during the pandemic (*no access – access*): 19% of those women and 11% of men decreased their childbearing plans (Table 3). Nevertheless, a substantial share of respondents who have gained access to HBW (*no access – access*) or had access to HBW already before the pandemic (*access – access(* +*)*) also increased their fertility intentions (10–12% of men and 7–13% of women).

**Table 1:** The share (in %) of respondents having access to home-based work before and during the Covid-19 pandemic

Date	Access to HBW	Mothers	Fathers	Total
February 2020	Yes	26.7	20.7	23.6
	No	73.3	79.3	76.4
	Sum	100	100	100
June 2021	Yes	41.9	32.5	37.0
	No	58.1	67.5	63.0
	Sum	100	100	100

Source: Own calculations based on Polish Familydemic Dataset

**Table 2:** The structure (in %) of respondents by fertility intentions, age, and gender in the Polish Familydemic Dataset

Sex	Age	Fertility intentions			Sum
		Hold the same	Decrease	Increase	
Mothers	20–24	62.96	22.22	14.81	100.00
	25–29	68.87	15.09	16.04	100.00
	30–34	72.98	16.53	10.48	100.00
	35–39	81.39	10.82	7.79	100.00
	40–44	93.18	3.98	2.84	100.00
	Total	78.53	12.39	9.08	100.00
Fathers	20–24	75.00	4.17	20.83	100.00
	25–29	70.24	13.10	16.67	100.00
	30–34	73.73	13.14	13.14	100.00
	35–39	79.12	9.16	11.72	100.00
	40–44	88.64	6.06	5.30	100.00
	Total	79.57	9.53	10.90	100.00

Source: Own calculations based on Polish Familydemic Dataset

**Table 3:** The structure (in %) of respondents by fertility intentions, access to and use of home-based work (HBW), and gender in the Polish Familydemic Dataset

Sex	Access to HBW	Fertility intentions			Sum
		Hold the same	Decrease	Increase	
Mothers	no access—no access	80.86	10.11	9.03	100.00
	no access—access	68.50	18.90	12.60	100.00
	access—access( +)	79.28	13.51	7.21	100.00
Fathers	no access—no access	80.90	8.34	10.76	100.00
	no access—access	78.70	11.11	10.19	100.00
	access—access	76.68	11.40	11.92	100.00

Source: Own calculations based on Polish Familydemic Dataset

### Regression

We estimated multinomial logit regressions with the dependent variable assuming three categories: increase in, decrease in or unchanged fertility intentions. We run our models by sex (women and men), accounting for the moderators and the control covariates (basic model). Next, we allow for interactions between our moderators and our main explanatory covariate, measured by change in access to and use of HBW. While interpreting our findings, we refer to predicted probabilities (estimated marginal means) rather than to odds ratios, as they are recommended as the most accurate and straightforward inference in multinomial regressions (Paolino, 2021, Wullf 2015). Estimated marginal means are interpreted as the predicted probability that the response (change in fertility intentions) takes a certain value (decrease, increase, hold the same) depending on the value of the selected explanatory covariate and averaged over all the remaining covariates. We evaluate whether the difference between two predicted probabilities is significant by comparing 83% confidence intervals. We do it following Austin and Hux (2002) who showed that two means differ from each other with the p-value at around 0.05 if 83% CI do not overlap. The estimates of our models can be found in the Appendix, Table 5.

#### Main Effects

**Fig. 1:**
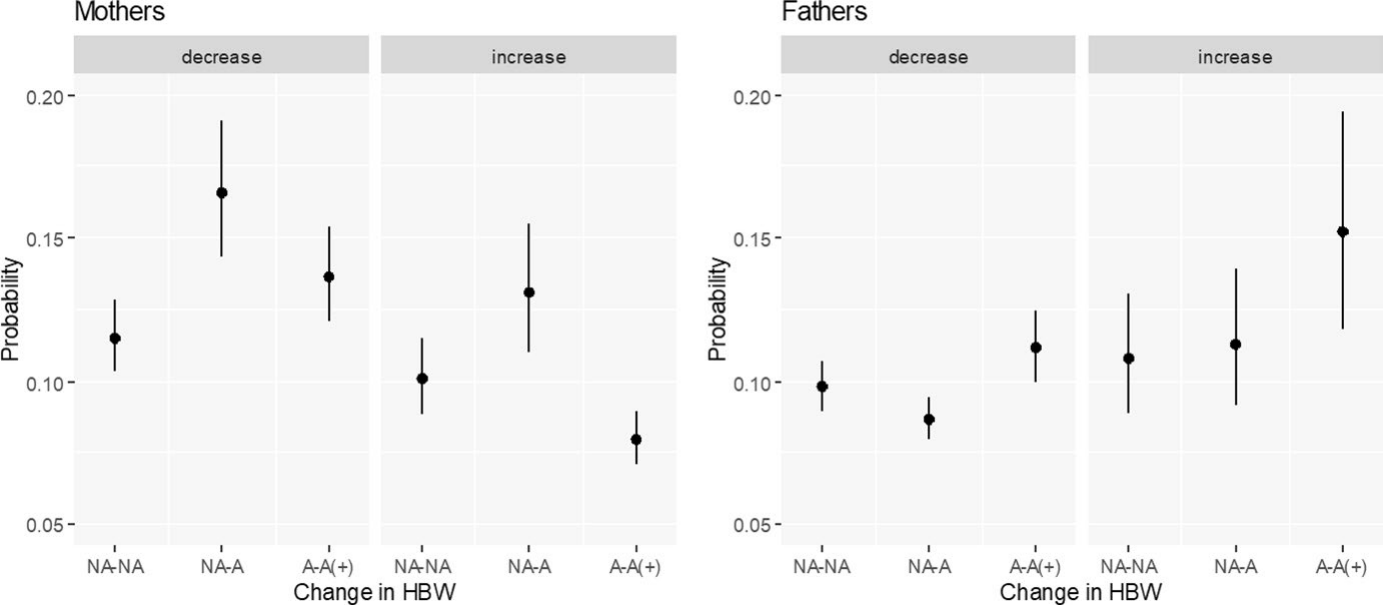
Predicted probabilities of decreasing and increasing fertility intentions by change in access to and use of HBW. Multinomial regressions by gender. The following categories of a change in access to home-based-work (HBW) between February 2020 and June 2021 are used: NA-NA—no access prior to the pandemic and no access in June 2021; NA-A—no access prior to the pandemic but access/use in June 2021; A-A( +)—continued access/use or intensified use of HBW. Source: Own calculations based on Polish Familydemic Dataset

Based on the estimates of basic models for mothers and fathers, we draw predicted probabilities (estimated marginal means) of a change in fertility intentions by change in access and use of HBW and present them with 83% confidence intervals in Fig. 1. We find partial support for our first hypothesis (H1) stating that the overall relationship between HBW and fertility intentions is negative, but only for women. Namely, gaining access to HBW during the pandemic (from no access to access; hereafter NA–A) seems to be related to a decline in further childbearing plans. This finding is manifested in a higher predicted probability of decreased FI in comparison to those mothers who did not have access to HBW before the pandemic and did not gain it (hereafter NA-NA; see Fig. 1, left-hand side). We do not find any statistically significant results for fathers.

#### Worsened Financial Situation

**Fig. 2:**
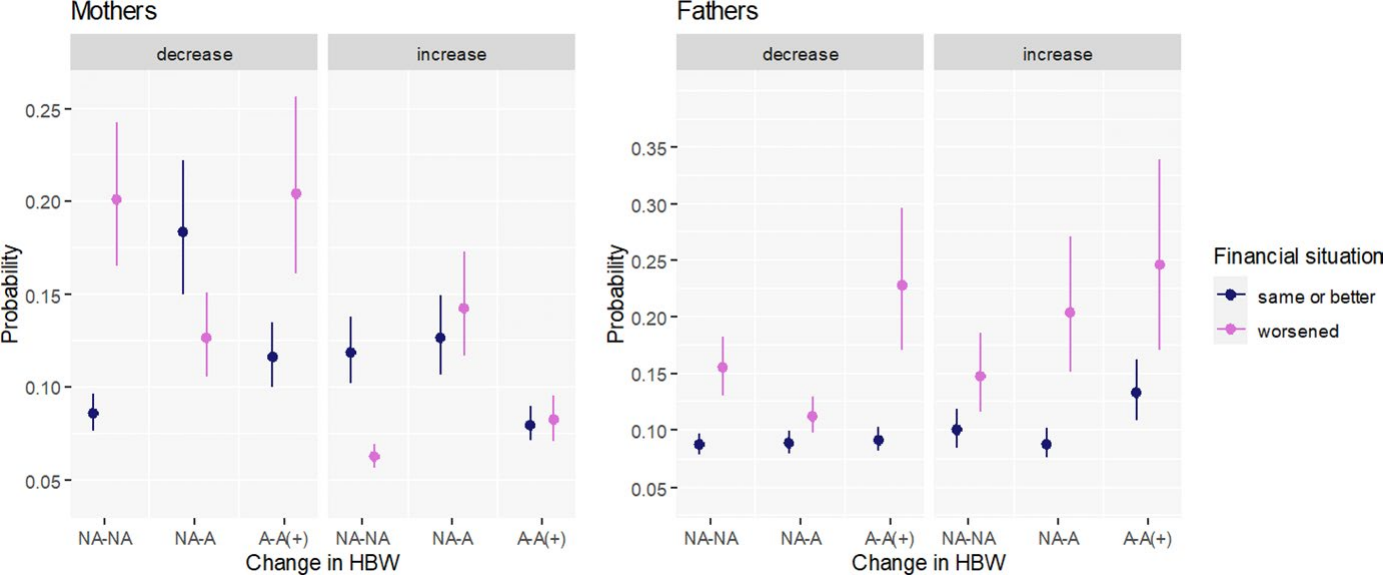
Predicted probabilities of decreasing and increasing fertility intentions by change in access and use of HBW and financial situation. Multinomial regressions by gender. The following categories of a change in access to home-based-work (HBW) between February 2020 and June 2021 are used: NA-NA—no access prior to the pandemic and no access in June 2021; NA-A—no access prior to the pandemic but access/use in June 2021; A-A( +)—continued access/use or intensified use of HBW. Source: Own calculations based on Polish Familydemic Dataset

Interactions with HBW bring some evidence that stays in line with our second hypothesis (H2) which expects that among parents whose financial situation has deteriorated during Covid-19 pandemic, HBW may bring important financial gains to cancel out or even outweigh the negative effects of HBW on fertility intentions. Among mothers whose financial situation worsened (Fig. 2) but who newly gained access to HBW (NA–A), the probability of increasing FI is higher than among those with worsened financial situation whose workplace arrangement has not changed (NA–NA). Furthermore, the newly gained access to HBW (NA–A) accompanied by worsened financial conditions of mothers and fathers is linked with a lower probability of decreasing FI than among on-site workers (NA–NA) (Fig. 2). Exactly the opposite (i.e., increase in the probability of a decline in FI) is observed among mothers whose financial situation remained the same or improved.

#### Childcare Burden Before the Pandemic

**Fig. 3:**
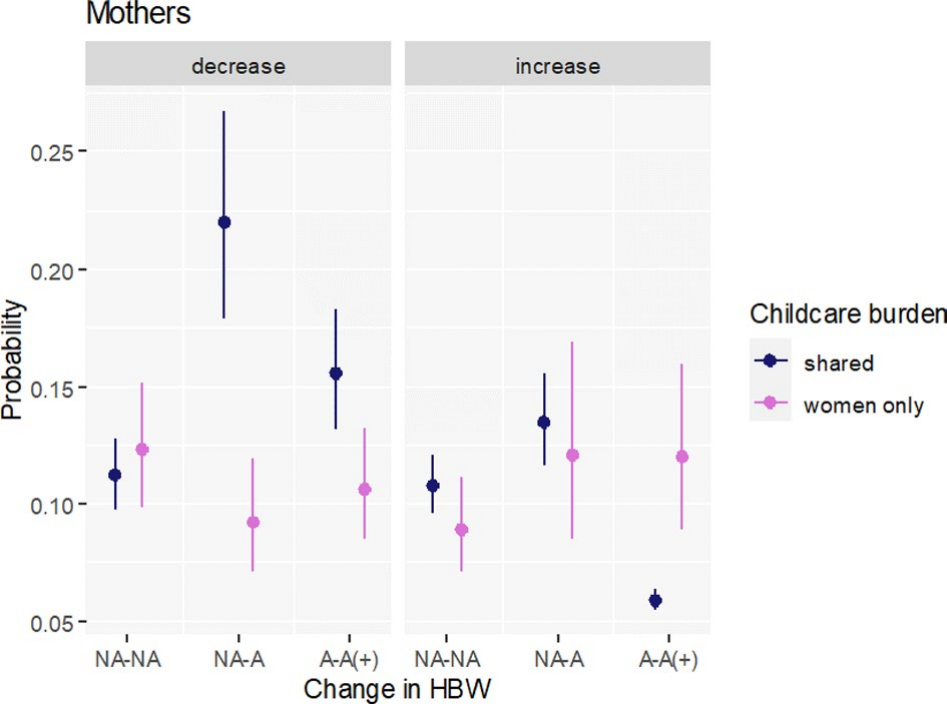
Predicted probabilities of decreasing and increasing fertility intentions by change in access and use of HBW and childcare burden. Multinomial regression for mothers. The following categories of a change in access to home-based-work (HBW) between February 2020 and June 2021 are used: NA-NA—no access prior to the pandemic and no access in June 2021; NA-A—no access prior to the pandemic but access/use in June 2021; A-A( +)—continued access/use or intensified use of HBW. Source: Own calculations based on Polish Familydemic Dataset

We have formulated two competing hypotheses concerning the moderating role of the division of childcare tasks between partners prior to the pandemic for fertility intentions of mothers. Hypothesis H3A states that the negative impact of HBW on fertility intentions during the pandemic vanishes among women living in egalitarian unions, i.e., who shared childcare more equally with their partners before the pandemic. Competing Hypothesis H3B posits that the negative impact of HBW on fertility intentions during the pandemic among women living in egalitarian unions would actually be stronger than among other women. Our findings support the competing hypothesis H3B. We show that for mothers in egalitarian relationships there is a negative link between HBW and change in fertility intentions and it actually vanishes out for women, who did most of or all of the childcare already before the pandemic. To be specific, those who shared childcare duties with their partners and had access to HBW already before the pandemic or who intensified its use (A–A( +)) are less likely to increase their childbearing plans than mothers with similar division of childcare having no access to HBW (NA-NA) (Fig. 3). Moreover, mothers in egalitarian unions who either gained access to HBW (NA–A) or had this access already / intensify its use (A–A( +)) are more likely to decrease their fertility plans than their egalitarian counterparts with no access to HBW (NA–NA).

#### Occupational Position

**Fig. 4:**
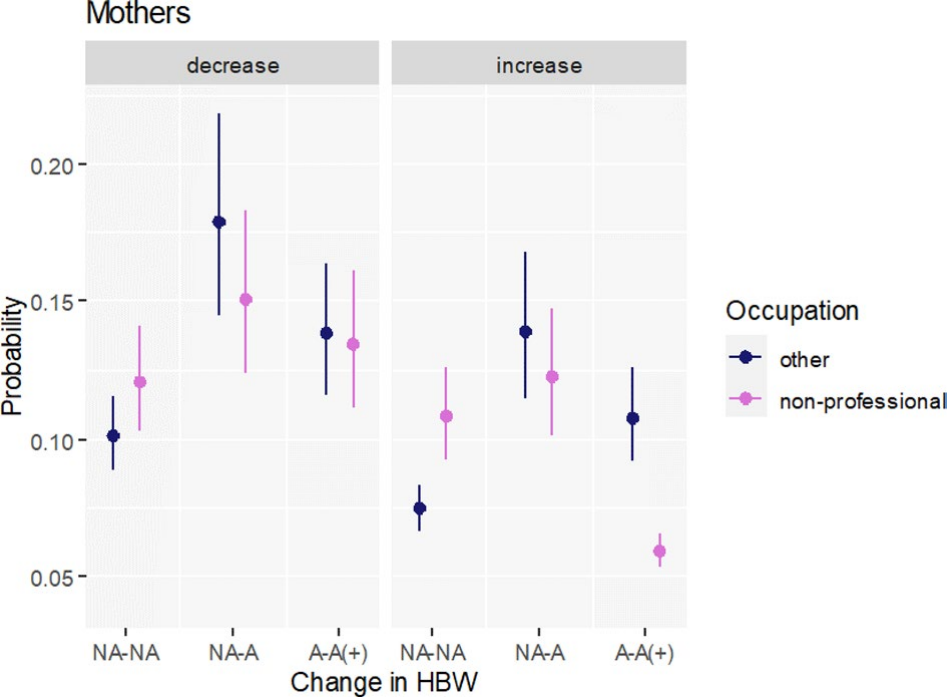
Predicted probabilities of decreasing and increasing fertility intentions by change in the access and use of HBW and occupational position. Multinomial regression for mothers. Note: The following categories of a change in access to home-based-work (HBW) between February 2020 and June 2021 are used: NA-NA—no access prior to the pandemic and no access in June 2021; NA-A—no access prior to the pandemic but access/use in June 2021; A-A( +)—continued access/use or intensified use of HBW. Source: Own calculations based on Polish Familydemic Dataset

Lastly, we expected (H4) that women holding non-professional/non-managerial positions who work from home are more likely to increase fertility intentions, as in this case they may be more willing to temporarily opt out from working actively. Our results do not support this hypothesis. Namely, we do not observe mothers holding non-professional occupations who gained access to HBW (NA-A) to have higher predicted probabilities of increased FI or lower predicted probabilities of decreased FI than women who neither had access nor gained access to HBW during the pandemic (NA-NA; Fig. 4). Furthermore, we do not find statistically significant differences between predicted probabilities of decreased FI among NA-A and A-A( +) groups. We, however, find that women holding non-professional occupations with continued access to HBW or intensified use of HBW are actually less likely to increase their FI compared to their counterparts with similar occupational positions but working from the office (NA–NA, Fig. 4; right-hand side).

## Discussion and Conclusion

In this study, we explored diverse aspects of working from home during the Covid-19 pandemic and their impact on fertility intentions among parents. As there may be both positive, as well as negative mechanisms linking home based work (HBW) and fertility intentions (FI), the overall effect depends on the circumstances. In our study, we argued that the Covid-19 pandemic-related lockdowns, school and childcare closures followed by widespread moves toward remote learning, even for the youngest children, as well as frequent individual and familial quarantaines created a situation in which the negative effects of HBW on the work-family reconciliation could have—on average—dominated the positive ones. Parents were faced with the need to simultaneously combine paid work and childcare or/and homeschooling at their homes. This resulted in an immense increase in unpaid tasks, work fragmentation, multitasking and mental-load (e.g., Hjalmsdottir et al., 2021, Raile et al., 2021). Limited possibilities of outsourcing not only childcare but also housework (due to confinement measures) created additional burden on families. Women were the ones to bear the most of it (see, e.g., Meraviglia & Dudka, 2021; Zamberlan et al., 2021; Manzo & Minello, 2020), but men have also increased their engagement in unpaid work during the pandemic (see, e.g., Derndorfer et al., 2021, Farre et al., 2021). These experiences have been shared by large parts of the population across all countries.

Our findings are partly in line with this general expectation. Namely, we found that mothers who gained access to HBW during the pandemic experienced a decline in their fertility intentions. Our study thus shows that the pandemic context has accentuated the role of negative aspects of HBW for combining work and care for mothers. This might explain why our findings are in contrast to the findings of Sinyavskaya and Billingsley (2015)—the only previous, published study on the topic—that found a positive relationship between access to HBW and FI in the pre-pandemic period in Russia.

We have also explored particular situations, in which we expected the negative relationship between HBW and FI to be leveled out or even outweighed by other (positive) mechanisms. First, we argued that HBW might have been perceived as a particularly convenient solution during the pandemic in cases when the family has suffered financially during this period. It has been shown that money savings have been one of the important advantages of working from home for people during the pandemic (Kučera et al., 2021, Rubin et al., 2020), and that financial savings from HBW were indeed substantial (Beno, 2021). Therefore, we expected that among individuals whose financial situation deteriorated during Covid-19 pandemic, HBW might have brought enough positive gains that canceled out or even outweighed the negative effects of HBW on fertility intentions during the pandemic. In our study, we found support for this expectation, as the effect was significant among both mothers and fathers.

Second, we considered two competing hypotheses on the role of pre-pandemic division of unpaid labor in couples. Our findings support the expectation that the negative effect of HBW on fertility intentions would be stronger among women, who shared childcare responsibilities with their partners before the pandemic. Specifically, we found that HBW had a negative effect on fertility intentions of mothers who shared childcare duties with their partners before the pandemic but not among those who already did most or all of the childcare duties before the pandemic. A possible explanation for this finding is that women who live in egalitarian or nearly egalitarian relationships may be used to the situation in which they share childcare duties equally with their partners or at least receive substantial support from them. They may even have more demanding jobs than other women, which simply do not allow them to spend much time or energy on childcare and/or housework, and the social distancing policies made it difficult for them to outsource the additional domestic and child-related duties. It is also likely that in a non-egalitarian context, such as the Polish one, men have not shared the increase in childcare and housework which emerged during the pandemic equally with their female partners, even if before the pandemic they participated in housework and childcare. In fact, numerous studies have shown that the additional housework and childcare burden, which emerged during the pandemic, was primarily carried out by women, regardless of whether they previously lived in egalitarian relationships or not (Hank & Steinbach, 2021; Manzo & Minello, 2020; Meraviglia & Dudka, 2021; Zamberlan et al., 2021) and regardless whether their male partners worked from home as well (Derndorfer et al., 2021). Mothers, from more traditional families in contrast to those from more egalitarian ones, might have been more used to the situation in which they have to carry out most of the childcare and housework and could more easily accept an increase in family-related responsibilities without questioning it (see, e.g., Sullivan & Lewis, 2001; Bailey & Kurland, 2002; Hilbrecht et al., 2008). For them the possibility to work from home could emerge as a convenient solution for combining paid work and care.

Finally, we also explored the situation of cumulation of negative effects of HBW on FI. We expected that for mothers holding jobs characterized by low autonomy, which are not suited to be executed from home, working from home while at the same time taking care of a child/children might have resulted in significant drop in their work satisfaction and—as a consequence—led to increased fertility intentions. The latter may serve as a way to temporarily opt out from working actively and to take advantage of generous maternity and parental leave entitlements in Poland, along with employment protection. Our findings do not confirm these expectations. We suppose that this is because employment and fertility plans of Polish women are also shaped by long-term, career- and earnings-related considerations. Namely, women holding lower occupational positions may be afraid to—even temporarily—opt out from working actively, regardless if they find their jobs unsatisfactory or not, as their earnings may be an important source of income for their families in the long-term. This finding may be particularly valid in the Polish context. It is because it is characterized by relatively low income levels and substantial contributions of women’s incomes to the household budgets (Klesment & Van Bavel, 2017; Osiewalska, 2019). But this finding may be increasingly relevant for other developed countries as well with the changing role of women in the society and increasing importance of women’s income in the family (Doepke et al., 2022; Vitali & Arpino, 2016).

All in all, our study demonstrates that HBW rather discouraged family expansion during the pandemic in the Polish context except for some specific social groups, i.e., women who were largely responsible for housework and childcare in addition to working for pay as well as households who had difficulties with making ends meet. These findings pertain to a very specific context, in which couples were unexpectedly faced with additional childcare obligations and were deprived of the possibilities to outsource some of the unpaid work. Nonetheless, they may also have important implications for the role of HBW on fertility in the aftermath of Covid-19 when HBW will likely be far more widespread than before the pandemic. Based on our findings, it can be concluded that having the possibility to work from home may encourage fertility of couples in worse financial situations for which this work arrangement implies important savings on work-related expenses. It may also facilitate family formation among couples with a more traditional division of labor in which she works for pay but is at the same time mostly responsible for the housework and childcare and is only a secondary income provider. For such women HBW may be an attractive possibility of combining paid work and care as they cannot count on the support from their partners. This implication may be particularly true in the country context like the Polish one where division of unpaid labor between partners is still heavily asymmetric and where male partners are very rarely responsible for larger share of housework or childcare and hardly ever take advantage of parental leaves (Kurowska et al., 2022). Different implications may be derived for women who are highly attached to the labor market. For these women, HBW may not constitute an attractive option for combining paid work and care, at least as long as it is utilized mainly by women, and not by men, and continues to exert negative consequences on career outcomes of home-based workers as well as increases their responsibility for childcare and housework as it used to happen before the pandemic (Sullivan & Lewis, 2001, Musch 2016). More research is needed, however, in order to establish how HBW affects fertility in different social contexts and what the mechanisms behind these effects are. So far, the topic has received little attention in demographic research.

This study has its limitations of which the most important are: the potential selection effects to employment and to HBW as well as some weaknesses of our measures of HBW. As for the first, some women and men may be more likely to be employed than others and some may be more eligible to HBW. These choices may relate to parenthood status and their subsequent fertility plans. Further, the pandemic hit some job sectors more than the others (e.g., service, sales) and working from home was also a solution for a limited number of workers. In order to reduce the selection bias, we control for SES which was shown to be an important determinant of one’s ability to HBW during the pandemic (Dingel & Neiman, 2020). Second, our main explanatory covariates, which defines whether the respondent received access to HBW during the pandemic, captures only two points in time: February 2020 and June 2021. We thus are not able to take into account potential changes in workplace arrangements that happened in between, e.g., we do not capture persons who gained access to HBW in mid-2020 but lost it half a year later.

Despite the limitations, our study provides an important contribution to literature on the complex interplay between work and family, shedding light on these relationships in the unprecedented times of the Covid-19 pandemic. It is the very first comprehensive study on the link between HBW and childbearing intentions, which not only provides novel empirical findings but also outlines a theoretical framework on how HBW may affect fertility intentions and behavior in the context of increased social uncertainty levels. It also helps to better understand the role of specific conditions for the direction of the link between HBW and fertility decisions. As such the study has a potential to stimulate future research, which will likely become widely discussed among demographers due to the growing body of literature pointing out numerous consequences of the pandemic on family development, including the role of uncertainty (e.g., Guetto et al., 2022), job and income loss (e.g., Luppi et al., 2020), termination of infertility treatment (e.g., Tippett, 2021) and access to external childcare (Aassve et al., 2020). With this study, we add another strand to this research by showing the importance of HBW for the change in fertility intentions during the pandemic.

## Figures and Tables

**Table 4:**
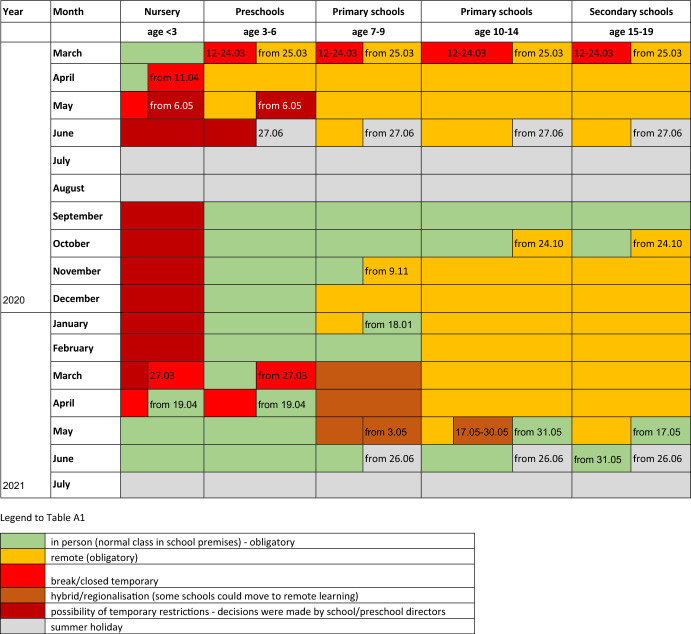
Confinement policies with respect to childcare and schooling in Poland in the studied period (March 2020 – June 2021)

## Data Availability

The dataset we are using is not yet publicly available. It comes from a CAWI survey carried out on a representative sample from the Polish population within the frames of the project funded by the National Science Center in Poland (PI—Anna Kurowska). After the end of the project the dataset is planned to be made available for other uses.

## Appendix

See Tables 4 and 5.

**Table 5:** Estimates (odds ratios) of multinomial logit models on the probability of a change in fertility intentions between February 2020 and June 2021

	MOTHERS								FATHERS				
	Model 1		Model 1 + interaction with worsened financial situation		Model 1 + interaction with non-professional or non-managerial position		Model 1 + Interaction with childcare burden		Model 1		Model 1 + interaction with worsened financial situation		Response
Predictors	OR	p-value	OR	p-value	OR	p-value	OR	p-value	OR	p-value	OR	p-value	
(Intercept)	0.066	** < 0.001**	0.057	** < 0.001**	0.054	** < 0.001**	0.060	** < 0.001**	0.186	** < 0.001**	0.191	** < 0.001**	Decrease
Age (ref. 30–34)													
20–24	2.387	**0.045**	2.339	0.052	2.443	**0.040**	2.376	**0.047**	0.220	0.157	0.217	0.152	Decrease
25–29	1.115	0.759	1.096	0.797	1.098	0.793	1.115	0.760	1.055	0.898	1.073	0.866	Decrease
35–39	0.649	0.148	0.609	0.100	0.651	0.153	0.649	0.148	0.740	0.339	0.763	0.391	Decrease
40–44	0.250	**0.002**	0.246	**0.002**	0.252	**0.003**	0.250	**0.002**	0.588	0.153	0.578	0.141	Decrease
Change in relationship status	0.947	0.946	0.925	0.923	0.977	0.977	0.977	0.977	0.427	0.449	0.407	0.426	Decrease
Insufficient housing conditions	0.891	0.769	0.949	0.895	0.878	0.742	0.913	0.816	0.808	0.577	0.805	0.572	Decrease
Worsened partnership quality	1.452	0.261	1.541	0.191	1.467	0.249	1.454	0.261	2.099	**0.023**	2.035	**0.032**	Decrease
Covid-19 health risk of any HH member	1.152	0.576	1.152	0.577	1.156	0.565	1.163	0.550	1.689	0.076	1.711	0.070	Decrease
Number of children	1.010	0.958	1.019	0.918	1.014	0.940	1.011	0.953	0.741	0.126	0.731	0.113	Decrease
Age of the youngest child (ref. 15-35 m)													Decrease
0-14 m	2.989	**0.002**	3.128	**0.002**	2.966	**0.002**	2.892	**0.003**	3.024	**0.005**	3.096	**0.004**	Decrease
3-6y	0.629	0.220	0.624	0.213	0.633	0.226	0.626	0.216	0.917	0.818	0.916	0.817	Decrease
7 + y	0.910	0.793	0.927	0.834	0.911	0.795	0.899	0.766	0.544	0.124	0.548	0.128	Decrease
High educational level	2.503	**0.002**	2.483	**0.002**	2.542	**0.002**	2.513	**0.002**	1.256	0.432	1.255	0.434	Decrease
Poor subjective financial situation	1.368	0.363	1.306	0.442	1.374	0.357	1.367	0.366	1.288	0.455	1.332	0.399	Decrease
Number of months partner worked from home	0.985	0.597	0.986	0.622	0.984	0.564	0.987	0.633	1.017	0.525	1.017	0.522	Decrease
Change in HBW (ref. No access—No access)													Decrease
No access—access	1.744	0.078	2.894	**0.004**	2.248	0.096	2.522	**0.036**	0.873	0.737	1.007	0.988	Decrease
Access—access ( +)	1.183	0.546	1.394	0.339	1.552	0.351	1.321	0.463	1.251	0.490	1.118	0.760	Decrease
Child care burden	0.943	0.392	0.926	0.274	0.944	0.409	1.001	0.993	0.847	0.095	0.847	0.094	Decrease
Worsened financial situation	1.909	**0.023**	2.904	**0.004**	1.890	**0.025**	1.871	**0.028**	2.346	**0.005**	2.206	**0.032**	Decrease
Non-professional or non-managerial position	1.052	0.845	1.034	0.897	1.331	0.482	1.061	0.818	0.815	0.476	0.808	0.458	Decrease
Interaction: Change in HBW & Worsened financial situation													Decrease
No access—access & Worsened fin sit			0.202	**0.020**							0.721	0.682	Decrease
Access—access ( +) & Worsened fin sit			0.709	0.548							1.862	0.375	Decrease
Interaction: Change in HBW & Non-prof. or non-manag. position													Decrease
No access—access & Non-prof./Non-manag. position					0.669	0.531							Decrease
Access—access ( +) & Non-prof./Non-manag. Position					0.664	0.483							Decrease
Inte raction: Change in HBW & Childcare burden													Decrease
No access—access & Childcare burden							0.809	0.244					Decrease
Access—access ( +) & Childcare burden							0.938	0.666					Decrease
(Intercept)	0.161	**0.004**	0.170	**0.005**	0.104	**0.002**	0.170	**0.006**	0.312	**0.025**	0.330	**0.033**	Increase
Age (ref. 30–34)													Increase
20–24	1.735	0.251	1.776	0.231	1.803	0.223	1.716	0.261	0.897	0.856	0.909	0.875	Increase
25–29	1.811	0.102	1.885	0.083	1.735	0.131	1.846	0.092	1.196	0.635	1.172	0.675	Increase
35–39	0.710	0.311	0.729	0.353	0.691	0.277	0.710	0.313	0.921	0.776	0.926	0.790	Increase
40–44	0.234	**0.005**	0.234	**0.005**	0.234	**0.005**	0.231	**0.005**	0.440	**0.027**	0.442	**0.028**	Increase
Change in relationship status	1.174	0.838	1.236	0.788	1.273	0.760	1.232	0.791	2.119	0.223	2.139	0.220	Increase
Insufficient housing conditions	0.583	0.285	0.578	0.280	0.554	0.243	0.604	0.320	1.082	0.820	1.049	0.892	Increase
Worsened partnership quality	2.457	**0.011**	2.409	**0.013**	2.535	**0.008**	2.484	**0.010**	1.815	0.059	1.865	0.051	Increase
Covid-19 health risk of any HH member	1.189	0.538	1.205	0.506	1.180	0.557	1.198	0.522	1.371	0.253	1.377	0.247	Increase
Number of children	0.761	0.201	0.759	0.197	0.769	0.221	0.757	0.193	0.592	**0.005**	0.591	**0.005**	Increase
Age of the youngest child (ref. 15-35 m)													Increase
0–14 m	1.236	0.659	1.196	0.709	1.258	0.633	1.274	0.615	0.936	0.865	0.913	0.817	Increase
3-6y	1.156	0.709	1.155	0.712	1.177	0.676	1.180	0.672	0.668	0.212	0.650	0.186	Increase
7 + y	1.092	0.824	1.091	0.827	1.126	0.766	1.125	0.769	0.479	**0.028**	0.475	**0.026**	Increase
High educational level	1.069	0.826	1.081	0.797	1.099	0.758	1.084	0.790	0.882	0.635	0.890	0.661	Increase
Poor subjective financial situation	1.126	0.772	1.150	0.734	1.119	0.784	1.101	0.815	0.858	0.659	0.861	0.668	Increase
Number of months partner worked from home	0.994	0.849	0.992	0.796	0.990	0.749	0.993	0.807	1.013	0.612	1.013	0.598	Increase
Change in HBW (ref. No access—No access)													Increase
No access—access	1.487	0.263	1.274	0.554	2.357	0.127	1.580	0.361	1.037	0.927	0.860	0.762	Increase
Access—access ( +)	0.783	0.463	0.664	0.278	1.613	0.387	0.543	0.202	1.537	0.173	1.395	0.334	Increase
Child care burden	1.003	0.972	1.007	0.927	1.005	0.945	0.959	0.670	1.191	**0.016**	1.191	**0.016**	Increase
Worsened financial situation	0.793	0.511	0.584	0.233	0.791	0.503	0.785	0.495	2.035	**0.015**	1.750	0.106	Increase
Non-professional or non-managerial position	0.968	0.911	0.962	0.895	1.581	0.314	0.962	0.894	1.002	0.994	0.998	0.994	Increase
Interaction: Change in HBW & Worsened financial situation													Increase
No access—access & Worsened fin sit			1.807	0.429							1.648	0.522	Increase
Access—access ( +) & Worsened fin sit			2.042	0.339							1.624	0.492	Increase
Interaction: Change in HBW & Non-prof. or non-manag. position													Increase
No access—access & Non-prof./Non-manag. position					0.525	0.372							Increase
Access—access ( +) & Non-prof./Non-manag. Position					0.319	0.105							Increase
Interaction: Change in HBW & Childcare burden													Increase
No access—access & Childcare burden							0.974	0.887					Increase
Access—access ( +) & Childcare burden							1.206	0.262					Increase
Observations	814		814		814		814		877		877		
R^2^ Nagelkerke	0.179		0.191		0.184		0.183		0.169		0.172		

For the bolded values of coefficients p < 0.05

Source: Own calculations based on Polish Familydemic Dataset
